# Supplementary material for: Content-rich biological network constructed by mining PubMed abstracts
Source: BMC Bioinformatics. 2004 Oct 8;5:147. doi: 10.1186/1471-2105-5-147 (PMC528731; doi:10.1186/1471-2105-5-147)
Supplement: Additional File 5 — The original Chilibot query results of the term "long-term potentiation (LTP)" and 22 other terms, limiting the latest references analyzed to the years 1990, 1995, 2000, and 2004. [file 1471-2105-5-147-S5.bz2 › chilibotAdditionalFile5/ltp2004/html/ERK_ATF.html]

 


 **ERK** and **ATF** 
  
Found 68 abstracts in PubMed,  **30 abstracts were retrieved and analyzed**.  


---

 Search Google  |
 PDF files only 
|  EDU domain only 

---

**Interactive relationship** (e.g. stimulation, inhibition, etc)

- **ERK**  activation by the PKA pathway was not required for CREB  **ATF**  1 phosphorylation but rather was necessary for CREB dependent up regulation of C EBPs expression.  Ref: 11682632 Mol Endocrinol, 2001
- In these cells, which express low levels of uPAR and malignancy, both  **ATF**  and EGF stimuli induced an interaction of the EGFR with uPAR and  **ERK**  activation.  Ref: 14720519 Exp Cell ResExp Cell Res, 2001

- :-)

  **Parallel relationship** (e.g. studied together, co-existance, homology, etc.)

  - In adult rats, ventilation with high pressure for 30 or 60 min did not affect arterial oxygenation, but resulted in enhanced phosphorylation of  **ERK**  1 2, JNK, c Jun, Elk 1 and  **ATF**  2 compared to normally ventilated rats.  Ref: 12412688 Eur Respir J, 2002
  - Kinase activity was measured with  **ATF**  2 and Elk 1 fusion proteins as substrates for p38 MAPK and  **ERK** , respectively, followed by Western blotting.  Ref: 12947314 Surgery, 2003
  - Cross linking of CD158j resulted in selective phosphorylation of c Jun NH 2 terminal protein kinase JNK and its upstream kinase, MKK4 that led to the expression of  **ATF**  2 and c Jun, all in the absence of extracellular signal regulated kinase  **ERK**  1 2 phosphorylation.  Ref: 12591902 J Exp Med, 2003
  - However, increased cytoplasmic expression of MAPK  **ERK**  P, SAPK JNK P, p38 P, CREB P, Elk 1 P, c Myc P,  **ATF**  2 P and c Jun P occurred in the region bordering the infarct core penumbra at 4 h following reperfusion.  Ref: 12677442 Acta Neuropathol Berl , 2003
  - Thrombin caused phosphorylation of p38 MAPK, its substrate  **ATF**  2, and JNK MAPK, but not  **ERK**  MAPK.  Ref: 12505871 Am J Physiol Heart Circ Physiol, 2003
  - Phospho specific antibodies for  **ERK** , JNK, RSK, CREB, and  **ATF**  2 were used to test the effects of PMI on specific proteins whose functioning are known to be regulated markedly by phosphorylation.  Ref: 12637955 Neuropsychopharmacology, 2003
  - Assays of DNA synthesis, cell proliferation, and migration were performed in response to sc uPA,  **ATF** , kringle, and CTF in the presence and absence of the plasmin inhibitors epsilon aminocaproic acid EACA and aprotinin, the Galphai inhibitor pertussis toxin, and the mitogen activated protein kinase 1 the upstream regulator of the extracellular signal regulated kinase  **ERK**  inhibitor PD98059.  Ref: 14718842 J Vasc Surg, 2004
